# Using Motivational Interviewing to Improve Parenting Skills and Prevent Problem Behavior During the Transition to Kindergarten

**DOI:** 10.1007/s11121-020-01102-w

**Published:** 2020-02-08

**Authors:** Elizabeth A. Stormshak, David DeGarmo, S. Andrew Garbacz, Laura Lee McIntyre, Allison Caruthers

**Affiliations:** 1 College of Education, University of Oregon, 5251, Eugene, OR 97403 USA; 2 Prevention Science Institute, University of Oregon, Eugene, OR USA; 3 University of Wisconsin–Madison, Madison, WI USA

**Keywords:** Child behavior, Family engagement, Motivational interviewing, Parenting skills

## Abstract

In this study, we examined the efficacy of a version of the Family Check-Up (FCU) adapted for kindergarten school entry with regard to parenting skills during the transition to school. We also examined whether improvements in parenting skills would mediate improvements in parent- and teacher-rated child behavior problems from kindergarten to second grade. The FCU is a motivational interviewing (MI) intervention designed to engage parents in treatment to improve parenting skills. Participants were parents of 365 children enrolled in one of five elementary schools in the Pacific Northwestern United States. Main and indirect effects were tested with structural equation path modeling using an intent-to-treat approach. The FCU was associated with improved change in parenting skills, and changes in parenting skills, in turn, predicted reductions in child behavior problems. Implications for embedding MI in family-centered interventions at kindergarten school entry are discussed. Trial registration: NCT02289092

It is well established that parenting skill interventions are the most effective treatments for improving child behavior problems (e.g., Kaminski et al. 2008). Randomized clinical trials that implement parenting skill training and then follow children longitudinally from infancy to adolescence have expanded our understanding of effective parenting skills at different developmental time points that lead to healthy child adjustment. This body of research provides one of the strongest arguments for the influential role that parents play in the development and maintenance of a range of problem behaviors throughout childhood and adolescence, and it is a key mediator in developmental models of risk that predict adverse outcomes for children (Dishion and Stormshak 2007). A systematic review of interventions conducted across a variety of cultures and socioeconomic groups indicates that parenting interventions are effective at enhancing positive parenting skills, reducing negative parenting and abuse, and increasing parenting knowledge and efficacy (Knerr et al. 2013).

In early childhood, parenting interventions that improve positive parenting, such as warmth and praise, have been shown to affect behavior over time for children as they enter school and as parents learn to support positive behavior in their children (Dishion et al. 2008; Kazdin 2005). Parenting interventions that reduce commands and harsh discipline predict fewer conduct problems at school entry, including ADHD symptoms (Daley et al. 2009; Webster-Stratton 1998). In each of these examples, longitudinal follow-up and comparison with a control group provide insight into the typical course of child development, the potential preventative impact of parenting interventions on behavior, and the role that effective parenting can play in reducing the risk of behavior problems and adjustment difficulties from early childhood to adolescence.

Despite these clear findings, much of the foundational research that supports parenting interventions was conducted several decades ago and used the format of parenting groups as the primary mode of delivery. Although many successful programs include group training (Brotman et al. 2016), problems such as attendance, staffing, attrition, and low dosage have proved to be barriers to implementing these programs in real-world communities (Stormshak et al. 2016). In response, individualized models of treatment were developed with the goal of reducing treatment time by providing brief, targeted interventions that motivate parents to make changes and build on strengths (Nock and Kazdin 2005). One goal of brief, tailored, adaptive approaches to intervention is to create sustainable intervention models that can be easily implemented in community settings, such as schools (Stormshak et al. 2016). The Family Check-Up (FCU) is one such intervention. The FCU is a brief, tailored intervention that incorporates motivational interviewing (MI), tailored feedback, and a menu of intervention options to support families in the process of change (Dishion and Stormshak 2007). The FCU has been demonstrated to be efficacious with a range of populations and in a variety of settings, including early childhood, middle school, and community mental health services (Dishion et al. 2008; Stormshak et al. 2018). School-based research using the FCU has focused on middle school youth and motivating parents to engage with their adolescents during this transition. However, no research has been conducted on the FCU as a tool for prevention of problem behavior at elementary school entry when parents are motivated to support school success (McIntyre et al. 2007).

When children transition to kindergarten, they are expected to have a number of school-readiness skills that enable them to learn in a public school environment. These skills include attention and behavioral regulation in the classroom, as well as social competencies. Longitudinal research indicates that children who do not enter school with these basic competencies are at increased risk for a variety of behavioral and adjustment problems. Poverty and family contextual stressors play a key role in limiting children’s language development and academic readiness at school entry (Yeung et al. 2002). Although during the past 10 years the rate of preschool attendance has increased, more children live in poverty, are raised by single parents, and experience challenges related to daily living conditions. Thus, although access to high-quality preschool may improve school readiness for children at risk, promoting parenting skills and reducing risk factors associated with poor home environments may be more beneficial in preparing children for kindergarten. Many children lack a basic foundation of school readiness; nearly half have difficulty and up to one-third have “some problems” during the kindergarten transition (Rimm-Kaufman et al. 2000.)

Despite the clear association between parenting and child outcomes at school entry, very few interventions in schools focus primarily on parents and emphasize family–school links (Reinke et al. 2009; Stormshak et al. 2010). The majority of school-based interventions for problem behavior focus on the individual child or on the school context (e.g., positive behavior support; Lewis et al. 2017). But because at school entry parents likely are motivated to help their children achieve at school, interventions that target families can capitalize on that motivation during this critical transition by providing additional support, especially around reading and learning at home (Bierman et al. 2015).

MI began as a treatment for substance abuse with a relatively simple foundation for delivery: telling people they have a problem is much less effective than motivating them to think about their problem and take responsibility for making changes themselves. MI has been used across a range of interventions to improve mental and physical health. For example, MI is quite common as a treatment for smoking and substance abuse and has been used to promote behavioral health and aid in obesity prevention (Borrelli et al. 2015; Jensen et al. 2011). A comprehensive review of MI skills and related outcomes suggests that therapist skills supporting change talk—or the client’s use of language that suggests motivation and willingness to change—were the best predictors of reductions in a range of health and risk behaviors, and that change talk is the strongest predictor of behavioral change over time (Magill et al. 2018).

When MI is applied to parenting interventions, results also support improved parenting skills and long-term behavioral adjustment. MI has been used to increase engagement in parenting interventions for low-income, diverse populations who are difficult to engage in treatment and is associated with attendance, participation in treatment, and long-term reductions in behavior problems (Nock and Kazdin 2005; Sibley et al. 2016; Winslow et al. 2016). MI has also been used with school-based mental health interventions as a way to increase collaboration between parents and schools and promote behavioral change (Frey et al. 2011). MI is central to the FCU, and therapists’ adherence to the FCU with the use of MI skills has been linked to client engagement and improved parenting, which, in turn, predicts improvements in child behavior (Smith et al. 2013; Smith et al. 2019).

In our study, we adapted the FCU for kindergarten school entry and tested its efficacy in terms of problem behavior over 3 years. We predicted that the FCU would be associated with improvements in parenting skills during the transition to school, which would, in turn, lead to improved child behavior. We randomly assigned 365 families with kindergarten children to receive the FCU at school entry or school as usual. We adapted the FCU for kindergarten to include assessments that specifically measure school readiness (reading skills, self-regulation) and parenting skills that target the family–school relationship at this age (communicating with your child’s school, home-to-school planning, reading with your child). We used MI to engage families in the FCU and subsequent treatment sessions, building on parent motivation and readiness to change while enhancing parents’ own skills, strengths, and behavior. We hypothesized that (a) parents randomly assigned to receive the FCU would show improved parenting skills during the transition to school, and (b) improved parenting skills would mediate improvements in parent- and teacher-rated behavior problems from kindergarten to second grade.

We tested an intent-to-treat (ITT) mediation hypothesis (Hayes and Rockwood 2017). Child adjustment factors are considered distal and mediated because the intervention is provided directly to parents and not to children; therefore, intervention effects on child adjustment should operate indirectly through the parenting behaviors. Mediation requires children in the FCU group to show greater declines in problem behaviors postintervention, represented as the direct effect of the intervention; parents in the FCU program would show greater pre–post improvements in parenting skill relative to parents in the control condition, and pre–post change in parenting behaviors would mediate any direct effect of the intervention on pre–post change in child behaviors. In ITT analyses, efficacy of a treatment relies on comparison of assigned groups regardless of dropout, variation in treatment implementation, variation in school contexts, or other unobserved factors that occur after random assignment, providing causal inference and unbiased estimates of intervention effects associated with group assignment in real-world conditions (DeGarmo and Gewirtz 2019).

## Method

### Participants and Setting

**Fig. 1:**
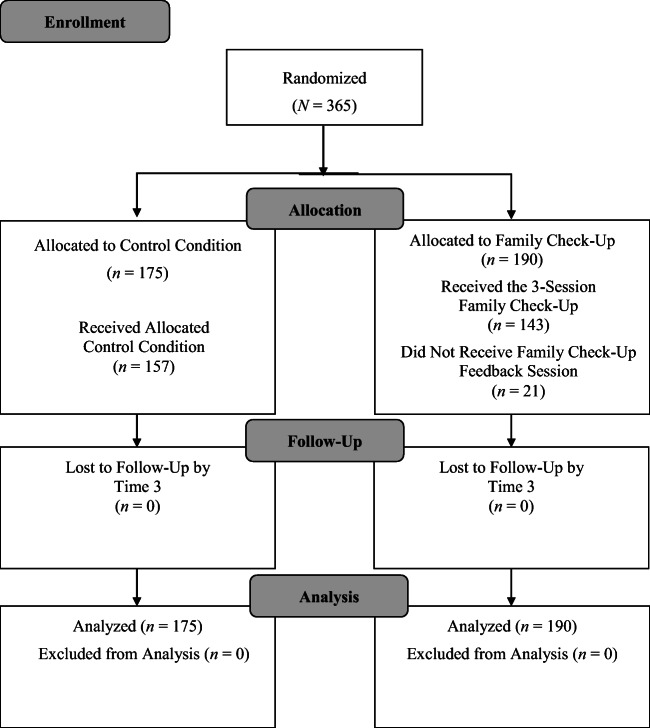
Participant enrollment

All families of kindergarten students across five elementary schools in an urban district in the Pacific Northwestern United States were invited to participate in this study during kindergarten registration. Four of the five schools were Title I schools, and approximately 65% of students across the five schools were eligible for free or reduced-price lunch. Average student enrollment at participating schools was 442 (SD = 98.94). Primary caregivers of 365 children provided consent. Across the five schools, 648 families had the opportunity to consent. Families who consented were randomly assigned by child sex to the FCU condition or a school-as-usual control condition (see Fig. 1) in a parallel design. Table 1 provides child demographic characteristics of the sample.

**Table 1:** Demographic characteristics of children

	% total (*N* = 365)	% FCU^a^ (*n* = 190)	% control (*n* = 175)
Mean (SD) child age	5.45 (0.50)	5.52 (0.50)	5.38 (0.49)
Child gender			
Female	45.8	45.7	47.7
Male	54.2	54.3	52.3
Child race/ethnicity			
White	58.9	59.18	58.0
Multiple races/ethnicities	22.1	20.7	23.6
Hispanic/Latino	13.4	13.4	13.4
Asian	2.2	1.8	2.5
Black/African-American	1.9	2.4	1.3
Unknown	1.2	1.8	0.6
Pacific Islander	0.3	0	0.6
Language child speaks at home			
English	88.8	90.2	87.3
Spanish	9.0	7.9	10.2
Other	2.2	1.8	2.5
Children received special school services^b^	14.5	14.7	14.3
Children attended preschool	60.3	52.6	68.6

^a^*FCU* Family Check-Up. ^b^Special school services may include a variety of services (e.g., behavior support plans)

On average, caregivers were age 33.90 years (SD = 6.32), approximately 89% of caregivers were female, and about 73% were White. Thirteen percent did not have a high school degree, 25% had a high school degree, 25% had completed some college coursework, 11% had a junior college or associate’s degree, 17% had a 4-year college degree, and 9% had graduate professional training or a graduate degree. Approximately 79% of caregivers lived with a spouse or partner. Their average gross annual household income ranged from $30,000 to $49,999.

### Measures

Caregivers and teachers reported about children’s behavior problems in the fall and spring of each year, starting during kindergarten (T1) through second grade (T3). Caregivers reported about their use of effective parenting strategies once each year. All measures were self-report and completed on paper-based questionnaires. These measures have been used extensively in our prior research, with adequate reliability and validity, as referenced below.

#### Child Behavior Problems

The latent child behavior problem variables were calculated from caregiver and teacher versions of the same measure at kindergarten and second grade: caregiver-reported concerns in fall and spring and teacher-reported concerns in fall and spring. Caregiver- and teacher-reported concerns of child behavior problems were assessed with nine items from parallel versions of the Strengths and Needs Assessment (Moore et al. 2016). Items addressed areas in which children may need additional support (e.g., behaves well; pays attention; sad, worried, or irritable; aggressive toward others). Items were rated on a 4-point scale ranging from 0 (*no concern*) to 3 (*serious concern*) and demonstrated strong internal consistency reliability (*α* = 0.944 and 0.933 for teachers fall and spring at T1, respectively, and 0.897 and 0.895 for parents at T1; *α* = 0.921 and 0.931, respectively, for teachers at T3, and 0.910 and 0.882 for parents).

#### Effective Parenting Strategies

Latent effective parenting strategies were calculated from four measures completed at kindergarten and second grade: limit setting, negative parenting, parent efficacy, and parent warmth. Limit setting was assessed with seven items from the Parenting Young Children Measure (McEachern et al. 2012) based on caregiver report of limit setting in the past month (e.g., make sure your child followed the rules that you set). Items that assessed limit setting were rated on a scale ranging from 1 (*never*) to 5 (*very often*). Limit-setting items had acceptable internal consistency reliability with the study sample (*α* = 0.786 and 0.785 at T1 and T3).

Negative parenting was assessed with five items from the Parenting Scale (Rhoades and O’Leary 2007) based on caregiver report of how often they had used negative parenting behaviors in the past month (e.g., you yelled or shouted at your child). Items that assessed negative parenting were rated on a scale ranging from 1 (*not at all*) to 5 (*very often*). Negative parenting items had acceptable alphas with the study sample (*α* = 0.702 and 0.670, at T1 and T3).

Parent self-efficacy was assessed with six items from the Behavioral Self-Efficacy Subscale of the Task Self-Efficacy Measure (Sanders and Woolley 2005). Caregivers rated their confidence for dealing with challenging child behaviors (e.g., your child argues with you about rules) on a scale ranging from 1 (*I could not deal with it*) to 10 (*I’m certain I could deal with it*). Caregiver self-efficacy items had strong internal consistency reliability with the sample (*α* = 0.925 and 0.933).

Parent warmth was assessed with five items from the Community Action for Successful Youth Measure (Metzler et al. 1998). Caregivers rated perceived warmth in their relationship with their child (e.g., if upset, this child seeks comfort from me) on a scale ranging from 1 (*definitely not*) to 5 (*definitely*). Items that assessed caregiver warmth had acceptable internal consistency reliability with the present sample (*α* = 0.794 and 0.772, at T1 and T3).

### Procedures

This study received approval from the authors’ institutional review board and is a clinical trial registered under the name, “The Positive Family Support Project: Partnering with Families for a Successful Transition to School” (NCT02289092). This study was implemented as a cluster randomized, controlled trial to test the efficacy of the FCU.

#### Family Check-Up Condition

The FCU uses a tiered approach to service delivery (Walker et al. 1996) in a specific scope and sequence for parenting support (Dishion and Stormshak 2007). The FCU consists of three steps and a menu of intervention services, each tailored for the individual family on the basis of their strengths, needs, and risk and delivered by FCU therapists. Families who agreed to engage in the intervention received an initial interview and ecological assessment during a single visit. Families were then offered a feedback session with goal planning. These sessions targeted areas most salient for kindergarten children and their families, including information about early learning, parenting skills, contextual stressors, home-to-school planning, and family strengths. The intake, assessment, and feedback session constitute completion of the FCU. Caregivers were offered a menu of intervention options, such as parent skill training and ecological management approaches (e.g., community referrals). Follow-up sessions were collaborative and included specific support in areas tailored for each family and based on goal-directed decisions made during the feedback session. The top five topics covered during meetings were (a) child behavior, (b) child academic skills, (c) positive parenting, (d) child emotional health, and (e) child peer relations. Of the 190 families in the FCU condition, 144 (76%) completed the initial meeting and assessment, 143 (75%) completed a feedback session, and 95 (50%) completed follow-up sessions, which were voluntary and based on the feedback and needs assessment. Throughout the FCU process, total treatment time averaged 161.22 min (range = 0.00 to 1120.00, accounting for some families who received no services). The average family in the intervention group received 4.36 total contacts (range = 0.00 to 26.00). At the conclusion of each contact, FCU therapists rated caregiver engagement during the session from 1 (*weak*) to 3 (*strong*), with an average of 2.81 (SD = 0.30).

#### Motivational Interviewing

MI is integrated throughout the FCU as the primary approach to guide parents’ decision-making. MI is used as a method of communication to promote individualized, goal-oriented change (Miller and Rollnick 1991). During the initial interview, FCU therapists glean information about caregivers’ motivation to change through a discussion about caregiver strengths, concerns, use of parenting strategies, and their child’s behavior. During the feedback session, therapists use MI to share information from the ecological assessment, develop a menu of intervention options, and guide parents’ decision-making to encourage motivation to change (Dishion and Stormshak 2007). MI is embedded in each follow-up session to promote goal-directed change based on each family’s strengths, needs, and goals.

#### Therapist Training

Therapists in this study were master’s- or doctoral-level psychologists previously trained in the FCU through a variety of means, such as attending a training workshop and working on prior projects that used the FCU. Training included FCU protocol, developmental norms, MI techniques, and academic supports for children in kindergarten (Stormshak and Dishion 2009). At the beginning of this project, therapists attended a full-day workshop on motivational interviewing delivered in the context of parent training and focused on key skills, such as using open-ended questions to elicit change talk and support goals. After training, therapists were required to observe three live FCUs (all three sessions: initial interview, ecological assessment, and feedback) and were subsequently observed leading two FCUs by a qualified supervisor. Before therapists were authorized to lead the FCU independently with study participants, they were required to meet criteria on two observed FCUs coded using the COACH rating system and were provided with support until they reached fidelity (Smith et al. 2013). The COACH is an observation system developed to measure adherence and fidelity to the FCU that includes five rating dimensions, including motivational techniques such as eliciting change talk and supporting. COACH ratings had to be within the satisfactory range (minimum score of 5 out of 9). Once therapists were providing the FCU independently, weekly group supervision meetings were held that emphasized case conceptualization and delivery of feedback to maximize treatment fidelity. Supervision was provided by licensed clinical psychologists with experience delivering the FCU. Continued fidelity was checked throughout the project using the COACH rating system which were required to be in the satisfactory range.

#### School-as-Usual Control Condition

Participants in the school-as-usual condition received traditional support from schools (e.g., behavior support plans) and support outside of school (e.g., mental health support). There were no significant differences between the FCU and school-as-usual conditions on the proportion of children who received special services in school, *χ*^2^ (1) = 0.308, *p* > 0.05, or those who received mental health services, *χ*^2^ (1) = 1.536, *p* > 0.05.

### Analytic Strategy

Results were examined using ITT analysis. The main efficacy hypotheses and indirect effects analyses were tested with structural equation path modeling (SEM) using Mplus 8.3 (Muthén and Muthén 1998–2019). SEM is a latent variable regression technique that simultaneously combines factor analyses with path analyses under the assumptions of multivariate normality. SEM models were specified as autoregressive change models, using the baseline kindergarten data (T1) and the postintervention second-grade data (T3). We specified across time error covariances for pre–post indicators as recommended for repeated measures and correlated error (Byrne 2011). Model fit was evaluated using recommended fit indices (Byrne 2011; McDonald and Ho 2002) of a chi-square minimization *p* value greater than 0.05, a comparative fit index (CFI) greater than 0.95, a chi-square ratio (*χ*^2^/*df*) less than 2.0, and a root mean square error of approximation (RMSEA) less than 0.08.

Efficacy hypotheses were tested with SEM specified as mediation analyses (MacKinnon 2008). Traditional mediation requires a direct intervention effect on the distal child adjustment outcome and on the proximal target of the intervention, parenting skill. Further, in the presence of a significant indirect effect from the intervention to parenting and parenting to child adjustment, the direct effect on child adjustment is rendered nonsignificant. More modern approaches focus squarely on the indirect effect of the intervention on the distal outcome through parenting as the putative mediating mechanism (Hayes and Rockwood 2017). To estimate indirect effects, the use of bias-corrected bootstrapped confidence intervals is recommended to allow distribution-free assumptions of the indirect effect (Preacher and Hayes 2008). Furthermore, modern approaches recommend full testing of the direct and indirect effects particularly when testing action theories or exploring conditions why there are no direct intervention effects (O’Rourke and MacKinnon 2018).

Clustering of children in classrooms and schools is a common design in education research and requires special consideration of nested data structure (Murray 1998; Raudenbush 1997). Although the FCU intervention trial was randomized at the student level, students were nested within five schools. In addition to using traditional single-level analyses, we also specified the primary analyses as multilevel SEM to examine the intraclass correlations and the design effect (DE) and to estimate a within- and between-school model to address the nonindependence of the students (Heck and Thomas 2015). For most common assumptions, multilevel modeling specification obtains results similar to those of single-level SEM (Curran et al. 2010; Heck and Thomas 2015). Prior commonly suggested criteria have included an intraclass correlation coefficient (ICC) smaller than 5%, which indicates multilevel modeling is unnecessary (Bliese 2000; ICCs ranged from 0.011 to 0.045). However, recent recommendations by Lai and Kwok (2015) suggest that when a DE exceeds 2.0, multilevel analyses are required to address DE bias in standard errors. The DE for a given variable is the ICC × (cluster *n* − 1) + 1. The DEs ranged from 1.58 to 4.24, with 80% larger than 2.0. Therefore, we conducted multilevel modeling with maximum likelihood with robust standard errors (MLR). SEM model fit was evaluated using recommended fit indices (Byrne 2011; McDonald and Ho 2002) of a chi-square minimization *p* value greater than 0.05, a CFI greater than 0.95, a chi-square ratio (*χ*^2^/*df*) less than 2.0, and an RMSEA less than 0.08.

Data were observed to be missing not at random (MNAR). Among the variables in the model, the only significant differences between complete and incomplete data cases were parent-reported limit setting scores. However, no differences were observed among the control variables or the parenting factor scores or child adjustment factor scores. We, therefore, used full information maximum likelihood (FIML), which uses all available information from the observed data in handling missing data. Even when data are MNAR, FIML can recover bias (Little et al. 2014), particularly with auxiliary variables that predict missingness in the model (Enders 2010).

## Results

Means, standard deviations, ICCs, and DEs for the key study variables are shown in Table 2. The average cluster size was 73 students per school, and the ICCs ranged from 0.01 to 0.05, typical of teacher- and parent-reported behavioral outcomes for young children. The majority of ICCs were less than 0.05; however, among the DEs, 11 of 16 possible DEs were greater than the 2.0 rule of thumb, or 69%. We, therefore, conducted analysis using both single-level and multilevel SEM path models. The primary hypothesis focused on whether the FCU had a direct effect on change in pre–post changes in child behavior problems and whether that effect was mediated by intervention-induced changes in pre–post parenting skill.

**Table 2:** Intraclass correlation and design effect for study outcome variables over time

	Time 1					Time 2				
	*N*	*M*	SD	ICC	DE	*N*	*M*	SD	ICC	DE
Limit setting	321	3.062	0.530	0.015	2.080	270	3.008	0.531	0.013	1.936
Negative parenting	320	0.869	0.550	0.017	2.224	270	0.862	0.508	0.017	2.224
Parent efficacy	318	4.341	0.745	0.013	1.936	269	3.854	0.871	0.018	2.296
Parent warmth	320	3.667	0.467	0.012	1.864	270	3.593	0.487	0.017	2.224
Teacher concerns, fall	244	5.602	6.803	0.019	2.440	262	5.454	6.304	0.017	2.224
Teacher concerns, spring	311	5.479	6.446	0.048	4.240	254	5.303	6.300	0.008	1.576
Parent concerns, fall	302	4.593	4.755	0.017	2.224	256	4.563	4.984	0.011	1.792
Parent concerns, spring	194	3.851	4.514	0.026	2.872	113	3.681	4.173	0.035	3.520

*Note. Time 1 = kindergarten and Time 2 = first grade. ICC*, intraclass correlation within school; *DE*, design effect calculated as ICC × (average cluster size − 1) + 1

**Fig. 2:**
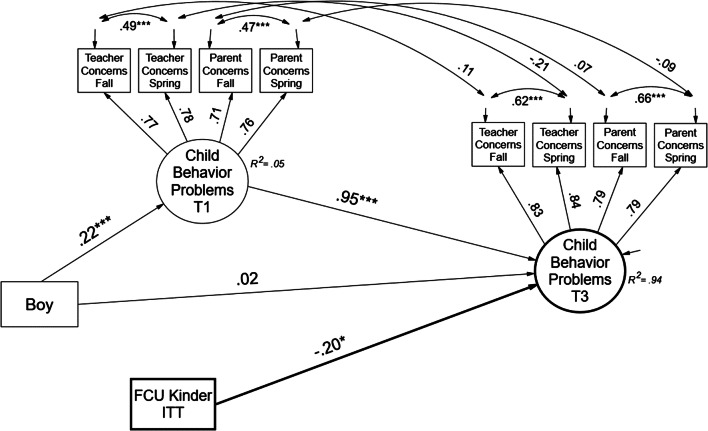
Structural equation path model for test of intent-to-treat (ITT) effect of FCU on pre–post child behavior problems. Paths are standardized multilevel within-group estimates. The ITT intervention contrast is *Y*-factor standardized to equal effect size. Model fit (*χ*^2^(52) = 256.23, *p* = 0.00, CFI = 0.90, RMSEA = 0.09; *χ*^2^/*df* = 4.92. ***p < 0.001; **p < 0.01; *p < 0.05). FTC effect size *d* = 0.20, a small effect. The direct effect is shown in bold. T1 = kindergarten and T3 = second grade

Overall, data supported the mediation hypothesis. The first step in evaluating mediation was a test of a direct effect of the FCU on the distal outcome of child adjustment problems. Findings are shown in Fig. 2 in the form of standardized path coefficients for the single-level estimates. The findings were substantively the same for the single-level and multilevel model, which accounted for clustering effects.

The FCU path was *Y* standardized to estimate the effect size (see Muthén and Muthén 1998–2019). After we controlled for biological sex, the FCU intervention predicted reductions in child behavior problems reported by teachers and parents (*β* = − 0.20, *p* < 0.05). A majority of the variance explained in the child behavior problems at second grade was accounted for by kindergarten levels; however, the FCU intervention accounted for a small effect in change variance (*d* = 0.20). The model obtained marginal fit to the data. Although the chi-square minimization *p* value was < 0.05, the model obtained a high CFI and an RMSEA near 0.08 (*χ*^2^(24) = 102.66, *p* = 0.00, CFI = 0.94, RMSEA = 0.09; *χ*^2^/*df* = 4.26). Teachers and parents reported higher levels of behavior problems at kindergarten for boys relative to girls (*β* = 0.22, *p* < 0.001) but not at second grade.

**Fig. 3:**
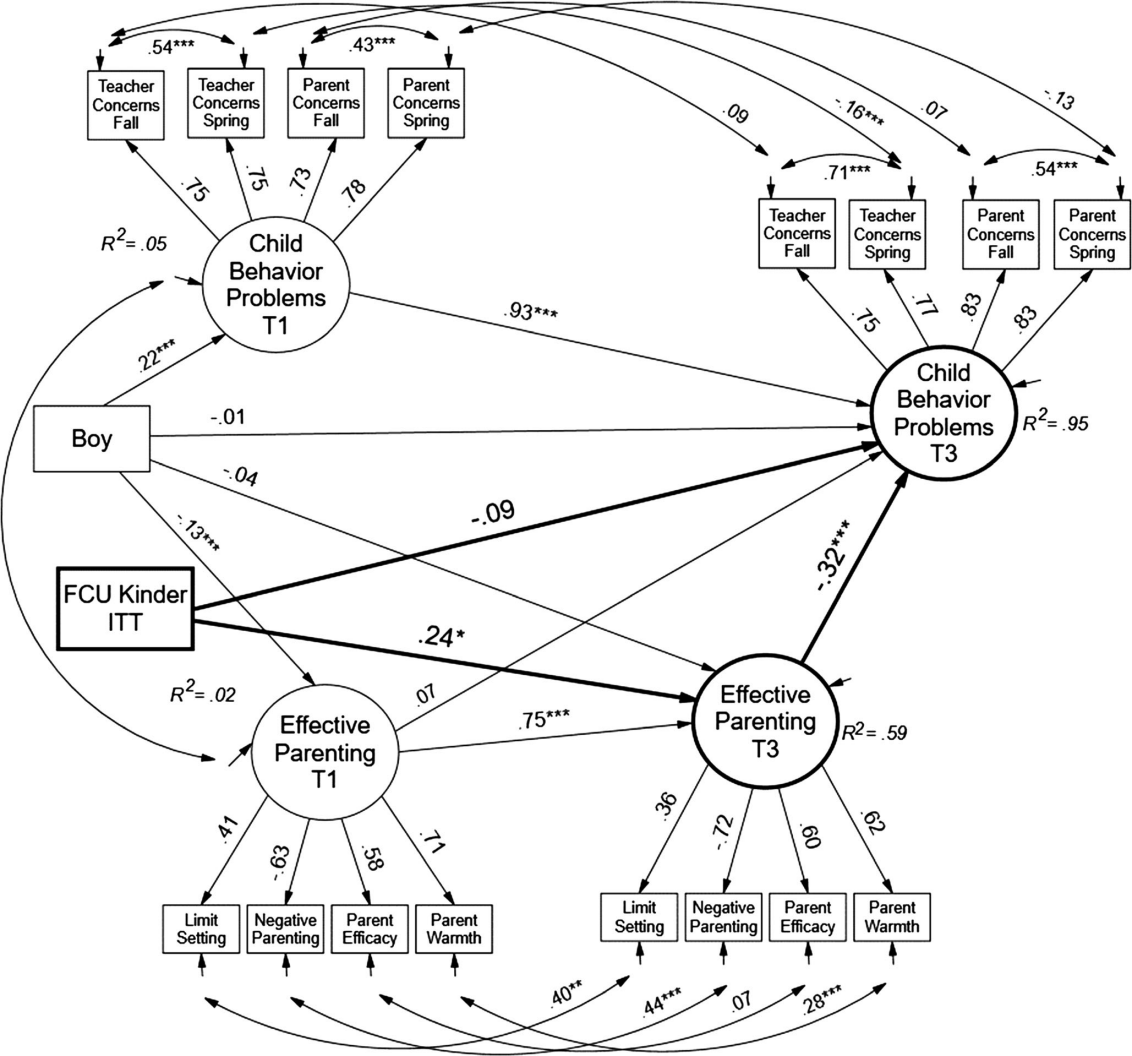
Structural equation path model for test of mediation hypothesis. Paths are standardized multilevel within-group estimates. The ITT intervention contrast is *Y*-factor standardized to equal effect size. Model fit (*χ*^2^(234) = 724.97, *p* = .00, CFI = 0.90, RMSEA = 0.07; *χ*^2^/*df* = 3.09. ***p < 0.001; **p < 0.01; *p < 0.05). FCU effect size on change in parenting, *d* = 0.24, a small effect. Bias-corrected bootstrapped *Y*-standardized indirect effect (FCU ➔ change parenting ➔change child problems) = − 0.07, *p* < 0.10, 95% CI (− 0.24, − 0.01), *d* = − 0.07. Primary mediation shown in bold. T1 = kindergarten and T3 = second grade

In the next step of the analyses, we tested mediation, including pre–post changes in parenting skill. Findings are shown in Fig. 3 in the form of standardized path coefficients for the single-level estimates. Again, the findings were substantively the same, accounting for the DEs in the data. Full mediation was partially supported. The model estimates showed that the direct effect of the FCU on child behaviors was rendered nonsignificant when changes in parenting were entered in the model. The FCU was associated with improvements in pre–post changes in parenting skill (*β* = 0.24, *p* < 0.05, *d* = 0.24, a small effect size). Effect size was reported as the *Y* standardized solution for the effect of a 0–1 dichotomous contrast, which is the mean intercept difference on the change factor divided by the standard deviation of *Y* (Muthén and Muthén 1998–2019). Finally, changes in parenting behaviors, in turn, predicted reductions in child behavior problems (*β* = − 0.32, *p* < 0.001). Boys were also associated with lower levels of parenting skill at baseline, relative to girls (*β* = − 0.13, *p* < 0.001, respectively).

We next estimated the indirect effect of the FCU through change in parenting to change in child behavior problems. Because Mplus does not bootstrap two-level analyses, we employed the R package RMediation by Tofighi and MacKinnon (2011) to bootstrap the indirect effect. The effect and confidence intervals were estimated with 1000 bootstrapped samples for the asymptotically distributed indirect effect. Partial support was obtained because the indirect effect was marginally significant. The standardized indirect effect was − 0.04, *p* < 0.05 (95% bootstrapped CI [− 0.085, − 0.005], *d* = − 0.08).

## Discussion

School entry is a critical developmental transition during which risk factors, such as poor behavioral regulation and attention skills, can affect long-term school success. Many parents are unaware of their child’s difficulties until school entry when children are required to demonstrate these skills in the classroom environment. As such, interventions that target at-risk families through parenting skill training should be delivered during these transitions to support child adjustment. Results of this study support the efficacy of the FCU delivered to parents at kindergarten entry in terms of the proximal outcome, improvements in parenting skills, and the distal outcome, improvement in child behavior. A strength of our measurement approach is that both parent and teacher reports were included in assessment of child behavior problems. Further, we demonstrate that there was evidence of partial mediation, with the direct effect rendered nonsignificant; however, the indirect effect was marginally significant. Because we adapted the FCU for a kindergarten sample and replicated prior evaluations of the intervention, we argue that directional hypotheses are appropriate. With directional hypotheses, there is support for full mediation in our model. That is, changes in parenting explain the association between the FCU at kindergarten transition and improvements in child behavior.

This prevention trial leveraged parents’ motivation to engage with their child and their school during the critical time of kindergarten transition. Through the use of MI, we encouraged all parents to become active participants in their child’s school readiness, emotional and behavioral health, and positive parenting and family routines. Parents engaged in the intervention at a high rate: 75% completed the FCU and 50% completed follow-up sessions. This level of engagement may be one factor that led to changes in parenting, although we did not factor dosage into our model. Parents’ engagement rate was higher than that of parents in prior FCU trials involving middle school youth (Stormshak et al. 2010), which suggests that at kindergarten transition, parents are motivated to improve their parenting skills, with a focus on school success. The FCU and associated MI components make this an ideal approach for prevention at this time.

### Limitations

An adaptation of the FCU for school use that reduces staff time and training is needed. Parent consultants were trained specialists who received weekly supervision and support in the context of our funded grant. We were able to train one community provider who worked with several families but then transitioned out of the position. Thus, it is unclear how schools can sustain this model of intervention. In addition, we used MI skills in each of our sessions with families but did not code for specific MI skills, instead focusing on general adherence to the model. Future research should examine the MI skills that were used with families, such as reflective listening and elicitation of change talk, and link the skills to changes in behavior.

### Implications

Efficacy findings for the FCU at kindergarten entry provide additional evidence in support of the FCU across developmental periods, building on a line of parenting skill intervention research (Kaminski et al. 2008). Schools may find it useful to proactively reach out to families when their child enrolls in kindergarten with strength-based, family-centered support through the FCU. This approach may strengthen existing school initiatives, such as positive behavior support (Lewis et al. 2017), by supporting parents in promoting goal-directed positive changes, creating home-to-school links, and establishing positive home–school routines in early elementary school (Moore et al. 2016). MI may be a core component school personnel can use to engage families as their child enters kindergarten to maximize their motivation during the transition to school. Research is needed that examines strategies to effectively support schools in adopting, implementing, and sustaining family-centered and home-to-school support in everyday practice. The FCU’s tiered framework is aligned with many common school initiatives, which may facilitate braiding an adapted FCU version with school-based initiatives (Stormshak et al. 2016).
